# (*E*)-Ethyl 2-cyano-3-(3,4-dihydr­oxy-5-nitro­phen­yl)acrylate

**DOI:** 10.1107/S1600536809035132

**Published:** 2009-09-05

**Authors:** Shi-Jie Zhang, Xian-Ming Zheng, Wei-Xiao Hu

**Affiliations:** a College of Pharmaceutical Science, Zhejiang University of Technology, Hangzhou 310032, People’s Republic of China; b Hisoar Pharmaceutical Co. Ltd, Taizhou 318000, Zhejiang Province, People’s Republic of China

## Abstract

The title compound, C_12_H_10_N_2_O_6_, was synthesized *via* a Knoevenagel condensation and crystallized from ethanol. In the crystal, strong classical inter­molecular O—H⋯O hydrogen bonds and weak C—H⋯N contacts link the mol­ecules into ribbons extending along [010]. Intra­molecular O—H⋯O and C—H⋯N contacts support the planar conformation of the mol­ecules (mean deivation 0.0270 Å).

## Related literature

For the syntheses of some potent and selective catechol *O*-methyl­transferase inhibitors, see: Bäckström *et al.* (1989 ▶). For structure–activity relationships of catechol *O*-methyl­transferase inhibitors, see: Tervo *et al.* (2003 ▶). For Entacapone-related crystal structures, see: Zheng *et al.* (2007 ▶). For the synthesis and anti­cancer evaluation of *E*-2-cyano-(3-substituted phen­yl)acyl­amides, see: Zhou *et al.* (2009 ▶).
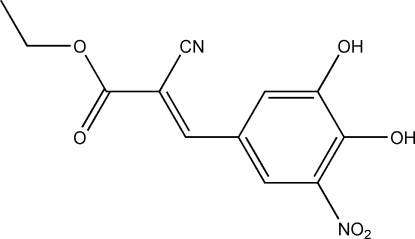


## Experimental

### 

#### Crystal data

C_12_H_10_N_2_O_6_
*M* *_r_* = 278.22
Monoclinic, 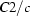

*a* = 24.983 (9) Å
*b* = 13.485 (5) Å
*c* = 7.312 (3) Å
β = 105.911 (4)°
*V* = 2369.0 (16) Å^3^
*Z* = 8
Mo *K*α radiation
μ = 0.13 mm^−1^
*T* = 93 K
0.40 × 0.20 × 0.10 mm

#### Data collection

Rigaku AFC10/Saturn724+ diffractometer
Absorption correction: none
9288 measured reflections
2714 independent reflections
2134 reflections with *I* > 2σ(*I*)
*R*_int_ = 0.042

#### Refinement

*R*[*F* ^2^ > 2σ(*F* ^2^)] = 0.048
*wR*(*F* ^2^) = 0.118
*S* = 1.00
2714 reflections
190 parameters
H atoms treated by a mixture of independent and constrained refinement
Δρ_max_ = 0.30 e Å^−3^
Δρ_min_ = −0.31 e Å^−3^

### 

Data collection: *CrystalClear* (Rigaku/MSC, 2008 ▶); cell refinement: *CrystalClear*; data reduction: *CrystalClear*; program(s) used to solve structure: *SHELXS97* (Sheldrick, 2008 ▶); program(s) used to refine structure: *SHELXL97* (Sheldrick, 2008 ▶); molecular graphics: *ORTEP-3* (Farrugia, 1997 ▶) and *PLATON* (Spek, 2009 ▶); software used to prepare material for publication: *publCIF* (Westrip, 2009 ▶).

## Supplementary Material

Crystal structure: contains datablocks I, global. DOI: 10.1107/S1600536809035132/si2196sup1.cif

Structure factors: contains datablocks I. DOI: 10.1107/S1600536809035132/si2196Isup2.hkl

Additional supplementary materials:  crystallographic information; 3D view; checkCIF report

## Figures and Tables

**Table 1:** Hydrogen-bond geometry (Å, °)

*D*—H⋯*A*	*D*—H	H⋯*A*	*D*⋯*A*	*D*—H⋯*A*
O1—H1*O*⋯O6^i^	0.91 (3)	1.76 (3)	2.6692 (18)	176 (2)
C5—H5⋯N1^ii^	0.95	2.57	3.467 (3)	159
O2—H2*O*⋯O3	0.96 (3)	1.72 (3)	2.584 (2)	149 (2)
C1—H1⋯N1	0.95	2.62	3.474 (3)	150

Symmetry codes: (i) 
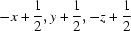
; (ii) 
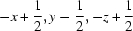
.

**Fig. 1.**
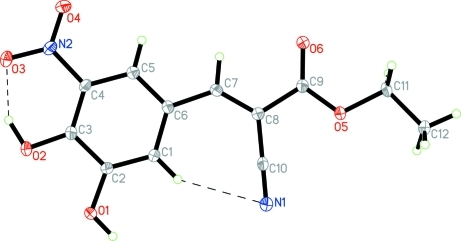
: The molecular structure of (I) shown with 50% probability displacement ellipsoids.

**Fig. 2.**
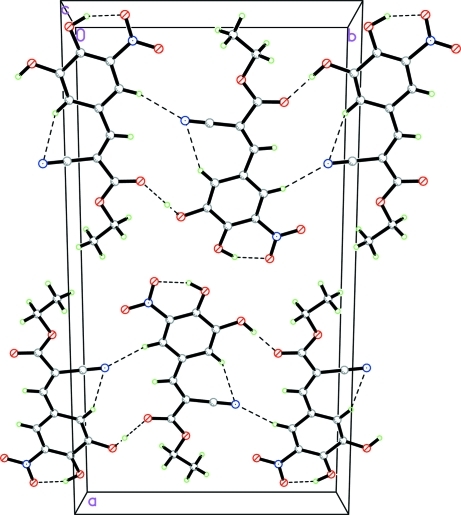
: A section of the crystal structure of (I), viewed down the c axis.

## supplementary crystallographic information

### Comment

Entacapone has been found to possess anticancer activity. Structure-activity relationships of entacapone revealed that catechol, cyano moieties and *trans* double-bond are necessary to sustain the activity and a nitro group substituted at C_5_ phenyl ring is preferable, and the amide group could be modified. (Bäckström *et al.*, 1989 & Tervo *et al.*, 2003) In continuation of our work on synthesis, crystal structure and anticancer evaluation of *E*-2-cyano-(3-substituted phenyl)acylamides, (Zheng *et al.*, 2007 & Zhou *et al.*, 2009) we synthesized *E*-2-cyano-substituted phenyl acrylic acid or its esters under Knoevenagel condensation, among which only *E*-2-cyano-3-(3,4-dihydroxyphenyl)acrylic acid had good *in vitro* KB inhibitory activity at IC_50_ 36 µ*M*. Herein, we present the structure of the title compound (I).

The molecular structure of (I) is illustrated in Fig. 1. The phenyl ring, atoms C7 > C9, O1 > O6 and N2 are almost coplanar, which makes dihedral angles of 14.2 (2)° and 8.72 (2)° with the planes C8/N10/C1 and O5/C11/C12, respectively. As shown in Fig. 2, the molecules are linked into chains along the *b* axis to form ribbons which are oriented parallel to the *a,b* plane. There are two intermolecular and two intramolecular (O—H···O and C1—H···N) hydrogen bonds (Table 1) which contribute to the formation of parallel ribbons in the crystal lattice.

### Experimental

To a stirred ethanol solution, was added 3,4-dihydroxy-5-nitrobenzaldehyde (4.9 g, 27 mmol), ethyl 2-cyanoacetate (3.4 g, 30 mmol) and ammonium acetate (0.75 g, 9.7 mmol). The mixture was heated to reflux for 6 h before filtration and the solid obtained was recrystallized from ethanol to afford the title compound as yellow solid, 6.1 g (81.9%); mp: 484–485 K; IR (KBr): 3446, 3232, 2223, 1687, 1602, 1543, 1284, 1221 cm^-1^; ^1^H NMR (DMSO-*d_6_*, 400 MHz) p.p.m.: 8.29, 8.10, 7.89, 4.32–4.28, 1.31–1.28; EIMS (%): 278 (*M*^+^, 17), 250 (31), 233 (23), 202 (55), 174 (31), 158 (34), 130 (25), 102 (28). The title compound was dissolved in ethanol and the solution evaporated gradually at r.t. to give single crystals of (I).

### Refinement

H atoms of the O—H groups were located from difference Fourier maps, they were freely refined. All H atoms of parent C atoms were placed in calculated positions and treated as riding, with C—H = 0.95 Å, and their displacement parameters set to *U*_iso_(H) = 1.2*U*_eq_(C).

### Crystal data

**Table:**

C_12_H_10_N_2_O_6_	*F*(000) = 1152
*M**_r_* = 278.22	*D*_x_ = 1.560 Mg m^−^^3^
Monoclinic, *C*2/*c*	Mo *K*α radiation, λ = 0.71073 Å
Hall symbol: -C 2yc	Cell parameters from 3450 reflections
*a* = 24.983 (9) Å	θ = 3.0–27.5°
*b* = 13.485 (5) Å	µ = 0.13 mm^−^^1^
*c* = 7.312 (3) Å	*T* = 93 K
β = 105.911 (4)°	Prism, yellow
*V* = 2369.0 (16) Å^3^	0.40 × 0.20 × 0.10 mm
*Z* = 8	

### Data collection

**Table:**

Rigaku AFC10/Saturn724+ diffractometer	2134 reflections with *I* > 2σ(*I*)
Radiation source: Rotating Anode	*R*_int_ = 0.042
graphite	θ_max_ = 27.6°, θ_min_ = 3.0°
Detector resolution: 28.5714 pixels mm^-1^	*h* = −28→32
multi–scan	*k* = −17→17
9288 measured reflections	*l* = −9→6
2714 independent reflections	

### Refinement

**Table:**

Refinement on *F*^2^	Primary atom site location: structure-invariant direct methods
Least-squares matrix: full	Secondary atom site location: difference Fourier map
*R*[*F*^2^ > 2σ(*F*^2^)] = 0.048	Hydrogen site location: inferred from neighbouring sites
*wR*(*F*^2^) = 0.118	H atoms treated by a mixture of independent and constrained refinement
*S* = 1.00	*w* = 1/[σ^2^(*F*_o_^2^) + (0.0596*P*)^2^ + 0.69*P*] where *P* = (*F*_o_^2^ + 2*F*_c_^2^)/3
2714 reflections	(Δ/σ)_max_ < 0.001
190 parameters	Δρ_max_ = 0.30 e Å^−^^3^
0 restraints	Δρ_min_ = −0.31 e Å^−^^3^

### Special details

**Table:**

Geometry. All e.s.d.'s (except the e.s.d. in the dihedral angle between two l.s. planes) are estimated using the full covariance matrix. The cell e.s.d.'s are taken into account individually in the estimation of e.s.d.'s in distances, angles and torsion angles; correlations between e.s.d.'s in cell parameters are only used when they are defined by crystal symmetry. An approximate (isotropic) treatment of cell e.s.d.'s is used for estimating e.s.d.'s involving l.s. planes.
Refinement. Refinement of *F*^2^ against ALL reflections. The weighted *R*-factor *wR* and goodness of fit *S* are based on *F*^2^, conventional *R*-factors *R* are based on *F*, with *F* set to zero for negative *F*^2^. The threshold expression of *F*^2^ > σ(*F*^2^) is used only for calculating *R*-factors(gt) *etc*. and is not relevant to the choice of reflections for refinement. *R*-factors based on *F*^2^ are statistically about twice as large as those based on *F*, and *R*- factors based on ALL data will be even larger.

### Fractional atomic coordinates and isotropic or equivalent isotropic displacement parameters (Å^2^)

**Table:**

	*x*	*y*	*z*	*U*_iso_*/*U*_eq_	
O1	0.39296 (5)	0.60992 (8)	0.12800 (18)	0.0219 (3)	
O2	0.45623 (5)	0.47578 (9)	0.04941 (17)	0.0222 (3)	
O3	0.47644 (5)	0.28865 (9)	0.03118 (18)	0.0269 (3)	
O4	0.41766 (5)	0.17833 (9)	0.07290 (19)	0.0292 (3)	
O5	0.13633 (5)	0.39925 (8)	0.35194 (16)	0.0196 (3)	
O6	0.17659 (5)	0.25177 (9)	0.32497 (17)	0.0251 (3)	
N1	0.20946 (6)	0.59192 (11)	0.2676 (2)	0.0237 (3)	
N2	0.43188 (6)	0.26508 (11)	0.0670 (2)	0.0222 (3)	
C1	0.32709 (7)	0.48910 (12)	0.1768 (2)	0.0179 (4)	
H1	0.3033	0.5391	0.2023	0.022*	
C2	0.37531 (7)	0.51576 (12)	0.1344 (2)	0.0174 (4)	
C3	0.41109 (7)	0.44238 (13)	0.0937 (2)	0.0180 (4)	
C4	0.39609 (7)	0.34321 (12)	0.1026 (2)	0.0178 (4)	
C5	0.34721 (7)	0.31586 (12)	0.1455 (2)	0.0189 (4)	
H5	0.3379	0.2477	0.1489	0.023*	
C6	0.31215 (7)	0.38778 (12)	0.1831 (2)	0.0177 (4)	
C7	0.26290 (7)	0.35205 (12)	0.2309 (2)	0.0186 (4)	
H7	0.2601	0.2819	0.2352	0.022*	
C8	0.22018 (7)	0.40137 (12)	0.2704 (2)	0.0182 (4)	
C9	0.17571 (7)	0.34244 (12)	0.3181 (2)	0.0188 (4)	
C10	0.21410 (7)	0.50688 (13)	0.2690 (2)	0.0189 (4)	
C11	0.09158 (7)	0.34963 (13)	0.4102 (2)	0.0216 (4)	
H11A	0.1072	0.3013	0.5129	0.026*	
H11B	0.0668	0.3138	0.3013	0.026*	
C12	0.05971 (7)	0.42919 (13)	0.4796 (3)	0.0248 (4)	
H12A	0.0840	0.4609	0.5927	0.030*	
H12B	0.0276	0.3995	0.5119	0.030*	
H12C	0.0467	0.4789	0.3795	0.030*	
H2O	0.4735 (10)	0.4161 (19)	0.024 (3)	0.055 (7)*	
H1O	0.3692 (11)	0.659 (2)	0.139 (3)	0.063 (8)*	

### Atomic displacement parameters (Å^2^)

**Table:**

	*U*^11^	*U*^22^	*U*^33^	*U*^12^	*U*^13^	*U*^23^
O1	0.0197 (6)	0.0149 (6)	0.0348 (7)	−0.0012 (5)	0.0136 (5)	0.0001 (5)
O2	0.0176 (6)	0.0218 (7)	0.0314 (7)	−0.0007 (5)	0.0138 (5)	0.0004 (5)
O3	0.0211 (7)	0.0268 (7)	0.0379 (7)	0.0035 (5)	0.0169 (6)	0.0005 (6)
O4	0.0312 (7)	0.0163 (6)	0.0449 (8)	0.0022 (5)	0.0184 (6)	−0.0011 (5)
O5	0.0177 (6)	0.0184 (6)	0.0259 (6)	−0.0003 (5)	0.0115 (5)	0.0015 (5)
O6	0.0243 (7)	0.0172 (6)	0.0395 (8)	−0.0008 (5)	0.0183 (6)	0.0000 (5)
N1	0.0206 (8)	0.0207 (8)	0.0323 (8)	0.0006 (6)	0.0117 (6)	0.0001 (6)
N2	0.0214 (8)	0.0199 (7)	0.0268 (8)	0.0047 (6)	0.0094 (6)	−0.0005 (6)
C1	0.0162 (8)	0.0175 (8)	0.0215 (8)	0.0014 (6)	0.0076 (7)	−0.0003 (6)
C2	0.0180 (9)	0.0144 (8)	0.0206 (8)	0.0010 (6)	0.0067 (7)	0.0008 (6)
C3	0.0154 (8)	0.0203 (8)	0.0195 (8)	0.0004 (6)	0.0068 (7)	0.0000 (6)
C4	0.0173 (8)	0.0164 (8)	0.0208 (8)	0.0039 (6)	0.0069 (7)	−0.0003 (6)
C5	0.0192 (8)	0.0176 (8)	0.0211 (8)	−0.0004 (7)	0.0074 (7)	−0.0004 (6)
C6	0.0169 (8)	0.0176 (8)	0.0198 (8)	−0.0014 (6)	0.0070 (7)	−0.0004 (6)
C7	0.0204 (9)	0.0157 (8)	0.0211 (8)	−0.0019 (6)	0.0081 (7)	0.0003 (6)
C8	0.0188 (8)	0.0170 (8)	0.0202 (8)	−0.0005 (6)	0.0076 (7)	−0.0003 (6)
C9	0.0183 (9)	0.0193 (8)	0.0206 (8)	−0.0007 (7)	0.0085 (7)	−0.0013 (7)
C10	0.0146 (8)	0.0216 (9)	0.0223 (8)	−0.0013 (7)	0.0082 (7)	−0.0005 (7)
C11	0.0194 (9)	0.0221 (9)	0.0274 (9)	−0.0044 (7)	0.0133 (7)	−0.0010 (7)
C12	0.0224 (10)	0.0227 (9)	0.0346 (10)	−0.0005 (7)	0.0167 (8)	−0.0016 (7)

### Geometric parameters (Å, °)

**Table:**

O1—C2	1.3491 (19)	C3—C4	1.395 (2)
O1—H1O	0.90 (3)	C4—C5	1.391 (2)
O2—C3	1.334 (2)	C5—C6	1.384 (2)
O2—H2O	0.95 (3)	C5—H5	0.9500
O3—N2	1.2528 (19)	C6—C7	1.450 (2)
O4—N2	1.2266 (19)	C7—C8	1.354 (2)
O5—C9	1.322 (2)	C7—H7	0.9500
O5—C11	1.463 (2)	C8—C10	1.431 (2)
O6—C9	1.224 (2)	C8—C9	1.484 (2)
N1—C10	1.152 (2)	C11—C12	1.505 (2)
N2—C4	1.451 (2)	C11—H11A	0.9900
C1—C2	1.372 (2)	C11—H11B	0.9900
C1—C6	1.420 (2)	C12—H12A	0.9800
C1—H1	0.9500	C12—H12B	0.9800
C2—C3	1.419 (2)	C12—H12C	0.9800
			
C2—O1—H1O	117.0 (17)	C1—C6—C7	125.12 (15)
C3—O2—H2O	102.7 (15)	C8—C7—C6	131.17 (16)
C9—O5—C11	117.16 (13)	C8—C7—H7	114.4
O4—N2—O3	122.12 (14)	C6—C7—H7	114.4
O4—N2—C4	119.20 (15)	C7—C8—C10	125.13 (15)
O3—N2—C4	118.68 (14)	C7—C8—C9	118.16 (16)
C2—C1—C6	120.95 (15)	C10—C8—C9	116.71 (15)
C2—C1—H1	119.5	O6—C9—O5	125.26 (15)
C6—C1—H1	119.5	O6—C9—C8	122.58 (15)
O1—C2—C1	124.76 (15)	O5—C9—C8	112.16 (15)
O1—C2—C3	114.74 (15)	N1—C10—C8	179.64 (18)
C1—C2—C3	120.49 (15)	O5—C11—C12	106.83 (14)
O2—C3—C4	126.20 (15)	O5—C11—H11A	110.4
O2—C3—C2	116.01 (15)	C12—C11—H11A	110.4
C4—C3—C2	117.79 (15)	O5—C11—H11B	110.4
C5—C4—C3	121.88 (15)	C12—C11—H11B	110.4
C5—C4—N2	118.04 (15)	H11A—C11—H11B	108.6
C3—C4—N2	120.08 (15)	C11—C12—H12A	109.5
C6—C5—C4	120.10 (15)	C11—C12—H12B	109.5
C6—C5—H5	119.9	H12A—C12—H12B	109.5
C4—C5—H5	119.9	C11—C12—H12C	109.5
C5—C6—C1	118.76 (15)	H12A—C12—H12C	109.5
C5—C6—C7	116.10 (15)	H12B—C12—H12C	109.5
			
C6—C1—C2—O1	−179.03 (15)	C4—C5—C6—C1	0.1 (2)
C6—C1—C2—C3	0.7 (2)	C4—C5—C6—C7	−178.53 (15)
O1—C2—C3—O2	−1.9 (2)	C2—C1—C6—C5	0.0 (2)
C1—C2—C3—O2	178.35 (14)	C2—C1—C6—C7	178.44 (15)
O1—C2—C3—C4	178.37 (14)	C5—C6—C7—C8	−178.25 (17)
C1—C2—C3—C4	−1.4 (2)	C1—C6—C7—C8	3.3 (3)
O2—C3—C4—C5	−178.26 (15)	C6—C7—C8—C10	1.4 (3)
C2—C3—C4—C5	1.4 (2)	C6—C7—C8—C9	−178.89 (16)
O2—C3—C4—N2	1.8 (3)	C11—O5—C9—O6	2.8 (2)
C2—C3—C4—N2	−178.46 (14)	C11—O5—C9—C8	−176.96 (13)
O4—N2—C4—C5	1.1 (2)	C7—C8—C9—O6	0.8 (3)
O3—N2—C4—C5	−178.91 (14)	C10—C8—C9—O6	−179.52 (16)
O4—N2—C4—C3	−179.01 (15)	C7—C8—C9—O5	−179.44 (14)
O3—N2—C4—C3	1.0 (2)	C10—C8—C9—O5	0.3 (2)
C3—C4—C5—C6	−0.8 (2)	C9—O5—C11—C12	168.12 (14)
N2—C4—C5—C6	179.10 (14)		

### Hydrogen-bond geometry (Å, °)

**Table:**

*D*—H···*A*	*D*—H	H···*A*	*D*···*A*	*D*—H···*A*
O1—H1O···O6^i^	0.91 (3)	1.76 (3)	2.6692 (18)	176 (2)
C5—H5···N1^ii^	0.95	2.57	3.467 (3)	159
O2—H2O···O3	0.96 (3)	1.72 (3)	2.584 (2)	149 (2)
C1—H1···N1	0.95	2.62	3.474 (3)	150

Symmetry codes: (i) −*x*+1/2, *y*+1/2, −*z*+1/2; (ii) −*x*+1/2, *y*−1/2, −*z*+1/2.
